# The significance of controlled conditions in lentiviral vector titration and in the use of multiplicity of infection (MOI) for predicting gene transfer events

**DOI:** 10.1186/1479-0556-2-6

**Published:** 2004-08-04

**Authors:** Bing Zhang, Pat Metharom, Howard Jullie, Kay AO Ellem, Geoff Cleghorn, Malcolm J West, Ming Q Wei

**Affiliations:** 1 Department of Medicine, University of Queensland, Prince Charles Hospital, Brisbane, AUSTRALIA; 2 Queensland Institute of Medical Research, Brisbane, AUSTRALIA; 3 Department of Paediatrics and Child Health, Royal Children's Hospital, Brisbane, AUSTRALIA

## Abstract

**Background:**

Although lentiviral vectors have been widely used for *in vitro *and *in vivo *gene therapy researches, there have been few studies systematically examining various conditions that may affect the determination of the number of viable vector particles in a vector preparation and the use of Multiplicity of Infection (MOI) as a parameter for the prediction of gene transfer events.

**Methods:**

Lentiviral vectors encoding a marker gene were packaged and supernatants concentrated. The number of viable vector particles was determined by *in vitro *transduction and fluorescent microscopy and FACs analyses. Various factors that may affect the transduction process, such as vector inoculum volume, target cell number and type, vector decay, variable vector – target cell contact and adsorption periods were studied. MOI between 0–32 was assessed on commonly used cell lines as well as a new cell line.

**Results:**

We demonstrated that the resulting values of lentiviral vector titre varied with changes of conditions in the transduction process, including inoculum volume of the vector, the type and number of target cells, vector stability and the length of period of the vector adsorption to target cells. Vector inoculum and the number of target cells determine the frequencies of gene transfer event, although not proportionally. Vector exposure time to target cells also influenced transduction results. Varying these parameters resulted in a greater than 50-fold differences in the vector titre from the same vector stock. Commonly used cell lines in vector titration were less sensitive to lentiviral vector-mediated gene transfer than a new cell line, FRL 19. Within 0–32 of MOI used transducing four different cell lines, the higher the MOI applied, the higher the efficiency of gene transfer obtained.

**Conclusion:**

Several variables in the transduction process affected in *in vitro *vector titration and resulted in vastly different values from the same vector stock, thus complicating the use of MOI for predicting gene transfer events. Commonly used target cell lines underestimated vector titre. However, within a certain range of MOI, it is possible that, if strictly controlled conditions are observed in the vector titration process, including the use of a sensitive cell line, such as FRL 19 for vector titration, lentivector-mediated gene transfer events could be predicted.

## Background

Multiplicity of infection (MOI) is a parameter that has been commonly used to predict viral infectivity in a population of target cells. With wild type viruses, an "infectious unit" refers to the smallest amount of virus capable of producing an infection in a susceptible cell. The titre of the original suspension is defined as the number of infectious units per unit volume of the preparation [1]. In the field of gene therapy where viral vectors are used for gene transfer, MOI was adopted to represent the ratio of input infectious units (titrated on the target cell line) to the number of cells available for transduction [2]. Ideally, there should be a simple linear relationship between the viral vector titre, its dilution, the volume of viral vector suspension used, and the proportion of cells infected, taking into account the probabilistic nature of the infective process when the number of viral vector particle approximates the number of cells. However, at present, the number of viable vector particles (or vector titre) in a given vector stock is determined by a vector-mediated transduction process, which is of a non-linear nature and can be influenced by various factors. If MOI is based on vector titre that is "variable", then MOI is complicated by all of the factors that influence vector titration and determination. Unfortunately, the extent of which is poorly understood.

Recently, lentivirus-based gene transfer vectors have been developed and have shown considerable promise for gene therapy research. It is evident that this vector system has several distinct advantages, and rapidly emerges as the vector of choice for *in vitro *and *in vivo *gene therapy studies [3,4]. Most current lentiviral vectors in use are based on Human Immunodeficiency Virus (HIV) type 1. A transient, three or four-component, HIV-1 based vector system consisting of one or two packaging constructs, a transfer vector and a Vesicular Stomatitis Virus G glycoprotein (VSV-G) envelope has recently been described and widely used [5-10]. Several reports have demonstrated that the HIV-based vectors effectively transduced dividing and non-dividing cells *in vitro *and *in vivo *including hematopoietic stem cells [7,11], terminally differentiated cells such as neurons [9], retinal photoreceptors [8], muscle, liver cells [5] and dendritic cells [12].

Other lentivectors, such as those based on the feline immunodeficiency virus (FIV) [13], equine infectious anaemia virus (EIAV) [14], caprine arthritis/encephalitis virus (CAEV) [15], Jembrana disease virus (JDV) [7], bovine immunodeficiency virus [16] and visna virus [17], are examples of recently developed non-primate lentiviral vectors that have also demonstrated efficient gene transfer to various types of cells.

Just as with Moloney murine leukaemia virus (MoMLV) based retroviral vectors, many variables could theoretically affect the measurement of infectivity of lentiviral vector particles, such as target cell type, number, cycle, other modulators of cell membrane ingredients, the time needed for vector uptake and vector viability/susceptibility, half life during the transduction process or even Brownian motion in which the vector makes way to the target cell [18]. In addition, the issue of particle variation within the population of artificially assembled vector "infectious" units could be a contributory factor to between-preparation variation in the predictability of their infectious behaviour. Arai *et al *(1999) found that the ratio of cells transduced with the VSV-G-pseudotyped retroviral vectors based on MoMLV correlated with the result predicted from a Poisson distribution [9]. Generally with retroviral vectors using an ecotrophic or amphotrophic envelope, MOI at 1–3 is commonly used and results in around 30% of cells being transduced. The efficiency of gene transfer reaches a plateau after this. Higher MOI may reduce the number of transduced cells [3,19]. However, with lentiviral vector-mediated gene transfer, experiments employing MOI even greater than 1000 have been explored [12]. The rational behind the usage has obviously distinguished lentiviral vector from MoMLV based retroviral vectors. Unfortunately, there are, at present, no data available as to how lentiviral vectors behave in an *in vitro *transduction process, and how the variables affect vector titre determination and MOI usage.

In this study, we characterised factors that influenced the *in vitro *vector titration process, including the number of target cells being transduced, total number of viral vector particles, inoculum volumes (well beyond the depth of relevance to diffusion), vector decay and the period of vector adsorption (and thus vector decay). We also examined the use of various MOIs on several commonly used cell lines and tried to establish the relationship of MOI with the efficiency of gene transfer.

## Methods

### Cell cultures

Cell lines used in this study were a fetal rat liver carcinoma cell line, FRL 19; a human embryonic kidney cell line, 293 and its derivative, 293T; and a murine embryonic fibroblast cell line, NIH 3T3. FRL 19 was maintained at 37°C in Ham and Dulbecco's modified Eagle's medium (1:1 ratio, DMEM; Life Technologies Inc) containing 2 mM glutamine, 4% Fetal Calf Serum (FCS), 100 U/mL penicillin and 100 μg/mL streptomycin, 1 μg of fungizone per ml (Ham and DMEM), 10^-7 ^M of insulin, and 10^-7 ^M of dexamethasone in a 5% CO_2 _incubator. All other cells were maintained at 37°C in DMEM containing 2 mM glutamine, 10% Fetal Calf Serum (FCS), 100 U/mL penicillin and 100 μg/mL streptomycin, similarly in a 5% CO_2 _incubator. 293, 293T and NIH3T3 were maintained in DMEM containing 10% FCS, 2 mM glutamine, 100 U/ml penicillin and 100 μg/ml streptomcycin at 37°C similarly in a 5% CO_2 _incubator. Cells were seeded at 5 × 10^5 ^on 10 cm or 7.5 × 10^5 ^on 15 cm plate and were at 70 – 80% confluence at the time of transfection or transduction.

### Viral vector production

Replication-defective retroviral particles were generated by transient co-transfection of 293T cells with the three plasmids (pHR' CMVGFP or pHIV-CSGFP, pCMVΔR8.2 pr pCMVΔR8.9 and pHCMV-G), using a CaPO_4 _precipitation method as we previously reported [21]. Briefly, 293T cells were grown on 10 cm plates to 70–80% confluence and co-transfected with 10 μg pHCMV-G, 10 μg pHR' CMVGFP or pHIV-CSGFP and 20 μg pCMVΔR8.2 or pCMVΔR8.9. The plasmid DNA was diluted into 250 mM CaCl_2 _in 1/10-TE buffer (1 mM Tris HCl, 0.1 mM EDTA, pH 7.6) in 0.5 ml before an equal volume of 2× HBS (140 mM NaCl, 1.5 mM Na_2_HPO_4_, 50 mM HEPES, pH 7.05) was added and mixed by gently bubbling air through the mixture for 1 min. The solution was then added drop-wise onto the cells (100 μl per 1 ml of culture media). The cell cultures were rinsed with PBS and given fresh media within 10–12 hr after initiating transfection. The medium was harvested 48 hr post-transfection, centrifuged at low speed to remove cell debris and filtered through a 0.45 μm filter. The supernatant was stored at 4°C no more than 24 hr before it was used for transduction.

### Ultracentrifugation

This was performed as reported previously [20,21]. Briefly, 30 mL of vector-producing cell (VPC) supernatant was added to each polypropylene ultra-centrifugation tube (6 × 30 mL), and ultracentrifuged at 50,000 g for 2 hr at 4°C on AH629 rotors in a Beckman refrigerated centrifuge. After centrifugation, the tubes were promptly removed and supernatant decanted. The viral pellet was resuspended in 0.6 mL of DMEM and stored at -20°C.

### *In vitro *transduction and determination of lentivector titre

This was performed as we previously reported [20]. Briefly, cultured 293T cells were seeded at 5 × 10^5 ^cells and transduced with serially diluted and concentrated viral vector stocks 16–18 hours after seeding when cells were about 70% confluent. For each transduction, 8 μg/mL of polybrene (Sigma) was included in the transducing inoculum. Forty-eight hours after transduction, EGFP positive fluorescent cells were counted using epifluorescent microscope (Nikon eclipse E600, Japan) with the fluorescein isothiocyanate (FITC) excitation-emission filter set at 470 nm. The viral vector titre was determined as the average number of EGFP positive cells per 20 1-mm^2 ^fields multiplied by a factor to account for dilution of the viral stock as well as plate size and thus total cell number. Alternatively, 48 hours after transduction, cells were harvested, resuspended and sent for FACs analyse at a local FACS facility (Queensland Institute of Medical Research, QIMR, Brisbane, Australia).

### Transduction – studies of target cell volume and number

293T cells at 1 × 10^3^, 3 × 10^4 ^or 1 × 10^5 ^per well were seeded in triplicate in 24-well plates. Transduction was performed with the same stock of viral supernatant using volumes of 100 μl, 300 μl and 1 ml for 2 hours in the presence of 10 μg/ml polybrene. After the incubation period the cells were washed with fresh growth medium twice and allowed to grow for 2 days before the cells were trypsinised and fixed with 2% formaldehyde + 0.2% glutaraldehyde in PBS. EGFP positive and total cell numbers were counted with a haemocytometer using epi-fluorescence microscopy.

### Transduction – studies of variable vector-cell contact and adsorption periods

293T cells were grown in 24-well plates to approximately 70% confluence. The cells were incubated with 500 μl of pHR' CMVLacZ supernatant for 10 min, 30 min, 1 hr, 2 hr, 4 hr and 17 hours. After the indicated incubation period the viral supernatant was removed and replaced with fresh media. Forty-eight hours post-transduction, cells were stained to check for the presence of LacZ with the following solution: 5 mM K_3_Fe(CN)_6_, 5 mM K_4_Fe(CN)_6_.3H_2_O, 2 mM MgSO_4_, and 1 mg/ml X-gal in PBS. Blue cells or colonies were counted as positive for gene transfer.

### Transduction – studies of vector decay

Cell-free viral vector-containing supernatant was incubated at 37°C for 30 min, 2 hr, 4 hr, or 24 hr prior to being used as the transducing medium (500 μl), with experimental samples in triplicate. 293T cell at 70% confluent cultures were exposed to the transducing media for 2 hours, after which the inoculum was removed and the cultures replenished with fresh media. Forty-eight hr post-transduction, cells were stained to check for the presence of LacZ with the X-gal solution. Blue cells/colonies were counted in 3 fields and the average used as the titre at that time point.

### Transduction – studies of MOI and transgene expression

293T cells were plated in a 10 cm plate at 1 × 10^5 ^cells/plate. Transduction was performed with viral vector stocks at a MOI of 2, 4, 8, 16 and 32 in the presence of 10 μg/ml polybrene (Sigma). Transduced cells were passaged every three days and EGFP positive cells sorted at a local FACS facility (QIMR, Brisbane, Australia).

### Flow cytometry

Flow cytometry analysis was performed to evaluate the expression of lentivirus vector-mediated gene transfer. Cells were washed with PBS, and then fixed with 1% paraformaldehyde before the analysis. Samples were analysed on a FACScan flow cytometry in QIMR.

## Results

### The inoculum volume of the vector and the number of target cells affect vector titre determination, but not proportionally

**Figure 1:**
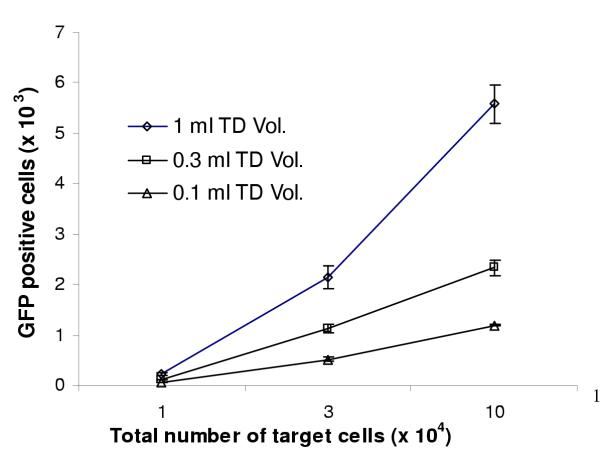
Higher inoculum volumes (more vector particles) and increased number of target cells resulted in higher efficiency of gene transfer. This was true over a range of target cells from 1 × 10^3 ^to 1 × 10^5 ^and volumes from 0.1 ml to 1 ml. However the increase in gene transfer was not proportional to the increase in inoculum volume. e.g. a 10 fold increase in volume resulted in only a 3.7 to 4.7 fold increase in transduction efficiency. The values represent mean ± SD (n = 4).

Figure 1 shows that during the lentiviral vector titration process, the higher the inoculum volume of the vector (ie. more viral vector particles) the more numbers of positively transduced cells. This was true over a range of target cells tested from 1 × 10^3 ^to 1 × 10^5 ^cells/ml. The results suggest the higher inoculum volume of the vector the more opportunity for a viral vector to reach a given target cell.

However, the results contradicted the data from an amphotropic MoMLV viral vector-mediated gene transfer where it was found that by keeping the virus vector concentration constant while the inoculum volume varied, the infectivity remained the same [19]. This discrepancy was not accounted for by the depth of fluid as in the present experiments, in the wells (area = 2 cm^2^) of the cell culture, the depth of the fluid varied from 0.5 mm (with a volume of 0.1 ml) to 1.5 mm for the 0.3 ml volume, and a depth of 5.0 mm for 1 ml of the vector preparation. All of these depths were well beyond the diffusion limit of relevance to the adsorption of 95% of a retrovirus preparation. This was because the rate decreased with the square of the depth, equating to 0.16 mm for a 2.5 hours adsorption period [2].

Similarly, vector titre was also affected by the number of target cells used in the vector titration process. A very significant increase in vector titre was noticed with increasing the cell numbers, but the increase was also not proportional. For a 30-fold increase in target cell number between 1 × 10^3 ^and 3 × 10^4 ^there was only an average of 9.17-fold increase in total number of transduced cells (for all transducing volumes). For a further 3.3-fold increase in cell number exposed in the same area, there was only a further 2.3 fold increase in total number of transduced cells. Thus, overall for a 100-fold increase in cell numbers (from 1 × 10^3 ^– 1 × 10^5^) exposed to vectors there was only a 21.3-fold increase in total number of transduced cells. Interestingly, the increase of the number of positively transduced cells was not proportional to the increase of the vector inoculum volume. The increase in the number of transduced cells was proportionally less than the increase in inoculum volume, e.g. a 10-fold increase in inoculum volume resulted in only a 3.7 to 4.7-fold increase in the number of positively transduced cells.

### Vector decay and the period of vector adsorption to target cells were significant factors in influencing the transduction process

**Figure 2:**
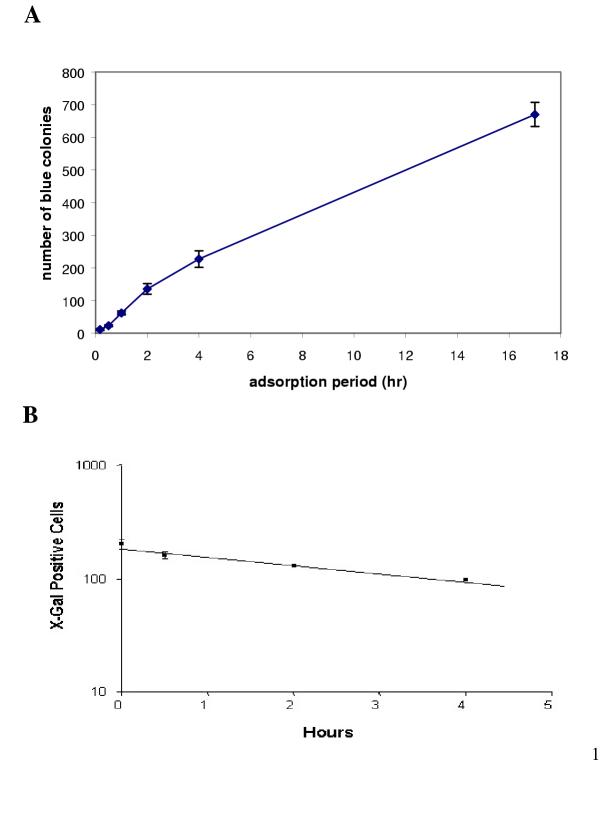
The period of adsorption (a) and vector decay (b) were significant factors in determining transduction efficiency. The duration of the adsorption period was also shown to alter the transduction efficiency significantly. As the incubation period increased so did the number of transduced cells. At 4 h less than half of the active vectors had adsorbed to the cells. To estimate the t_(1/2) _of the vector system used here, we pre-incubated the inoculum for increasing periods of time before applying aliquots to the target cell monolayer. By applying the following equations V^A ^= V^A^_o _exp (-k_d_t) and t_(1/2) _= ln(2)/k_d _to the data, {where V^A ^is the concentration of active virus at time t, V^A^_O _is the initial concentration of active virus, and K_d _is the virus decay rate constant}, the half-life of the vector was in the 8–9 hr range. The values represent mean ± SD (n = 4).

The length of period of vector adsorption to target cells was shown to alter the transduction efficiency significantly. As the incubation period increased so did the number of transduced cells (Figure 2a). At 4 hours less than half of the active vectors had adsorbed on to the cells. Since vector adsorption to cells was often protracted, the issue of thermostability of the vector preparation arose as a negative modulator of transduction efficiency with increasing time, thereby producing further variation in the estimated titre and thus the "MOI".

To estimate the half time (t_(1/2)_) of the vector system used here, we pre-incubated the inoculum for increasing periods of time before applying aliquots to the target cell monolayer for vector titre determination. The length of time for which the viral supernatant harvest was left at 37°C (in a cell-free environment) prior to use, noticeably affected the value of the vector titre (Figure 2b). The viral vector activity decayed logarithmically with time. By applying the following equations: V^A ^= V^A^_o _exp (-k_d_t) and t_(1/2) _= ln(2)/k_d _to the data, {where V^A ^is the concentration of active virus at time t, V^A^_O _is the initial concentration of active virus, and K_d _is the virus decay rate constant}, the half-life of the vector was in the 8–9 hours range. This is the first time that lentivector stability has been examined. This estimation was twice as long as that for wild-type HIV [1], suggesting that lentivector is much more stable.

### Variations in viral vector titration further complicated the use of MOI for predicting gene transfer events

Lentiviral vector titre (transducing unit per millitre, TU/ml) was calculated using the number of TU/ml times the dilution factor of the vector stock, divided by the volume of vector used in the transduction. As shown in the above results, the number of positively transduced cells changed when the transduction conditions varied. Therefore, the vector titre was affected by inoculum volume, vector stability and target cell numbers. If vector titres were to be calculated using the existing formula that was developed based on retroviral vector-mediated gene transfer, i.e. EGFP-positive cells (TU) ÷ volume of vector inoculum (ml), the titre of the original vector suspension would result in absurdly different figures (see Table 1), with ranges from 2.2 × 10^2 ^TU/mL to 1.2 × 10^4 ^TU/mL for the same viral suspension, more than a 50 fold difference. Likewise, because MOI is based on vector titre (MOI = titre × TD volume / number of cells), the use of MOI was thus affected.

**Table 1:** Different titres and MOI were obtained for the same vector stock when different numbers of target cells and volumes of inoculum were used. The number of positively transduced cells and thus the transduction efficiency, was also affected by the number of target cells in the transduction process, eg.: a thirty-fold increase in cell numbers resulted in a 53% decrease in efficiency. The transduction efficiency was highest with the smallest cell number and largest inoculum volume.

**Titre TU/mL (followed by MOI)**			Number of target cells
1 mL of VI Vol.	0.3 mL of VI Vol.	0.1 mL of VI Vol.	
2.24 × 10^2 ^(**0.224**)	3.96 × 10^2 ^(**0.119**)	6.08 × 10^2 ^(**0.061**)	1 × 10^3^
2.14 × 10^3 ^(**0.071**)	3.77 × 10^3 ^(**0.038**)	5.14 × 10^3 ^**(0.017**)	3 × 10^4^
5.58 × 10^3 ^(**0.056**)	7.79 × 10^3 ^(**0.023**)	1.19 × 10^4 ^(**0.012**)	1 × 10^5^

TU – Transducing Unit; VI Vol – Volume of Inoculum.

### Considerable differences existed in the sensitivity of lentiviral vector-mediated gene transfer in several conventional cell lines

**Figure 3:**
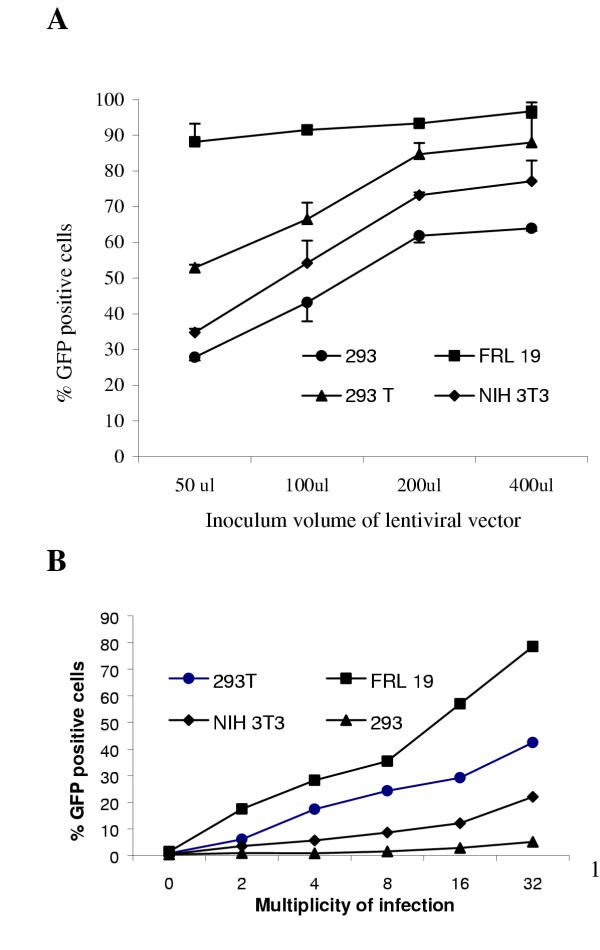
Efficiency of lentivector-mediated gene transfer to commonly used target cell lines (A) under different MOI (B). Four cell lines were seeded at 5 × 10^4^/well in 12 well plates. Several different inoculum volumes of lentivectors without known titre (A) or with known titre, ie.: different MOI (B) were added were added to each well (A) or as indicated. The media was changed daily. Cells were harvested three days after transduction, and washed three times with PBS. Transduction efficiency of lentivectors in different cell lines was obtained using flow cytometric analysis. Data represents mean value ± SD (n = 4).

The sensitivity of lentivector-mediated EGFP gene transfer to commonly used target cell lines has never been directly compared previously. In this study, 3 commonly used cell lines plus a new cell line FRL-19 were included for comparison. All cells were seeded in 12 well plate at 5 × 10^4 ^cells/well 16–18 hours before transduction. Concentrated viral vectors with unknown titre were added to each well at 50 μl, 100 μl, 200 μl, and 400 μl. Medium was changed every day. All cells were harvested 72 hours after transduction, washed twice with PBS, and then analysed by FACS. Figure 3 showed that the percentage of EGFP positive cells was 88.1% for FRL-19 cells, 52.9% for 293T cells, 34.7% for NIH 3T3 cells, and 27.8% for 293 cells respectively when 50 μl viral vector was used for transduction. Clearly transduction efficiency of lentivector-mediated EGFP gene transfer to FRL-19 was the highest amongst the four cell lines tested. It reached 96.7% when 400 μl of viral vector was used while the transduction efficiency of lentivectors was only 87.9% for 293T cells, 77.1% for NIH 3T3 cells, and 63.9% for 293 cells for the same volume of vector (Fig. 3A). When a third generation of lentiviral vector packaging system (pMDg/p, pRSV-Rev, gifts from Professor Didier Trono, Department of Genetics and Microbiology, CMU., Switzerland) were used to package a HIVCS-CMV-EGFP vector, a very similar transduction efficiency was obtained (Zhang et al., unpublished data). These results convincingly demonstrated that conventional cell lines were less sensitive to lentiviral vector-mediated gene transfer than FRL19, thus grossly underestimating vector titre.

### The sensitivity of cell lines to lentivectors was generally MOI dependent

We further examined whether the sensitivity of these cell lines to lentivectors-mediated EGFP gene transfer was dependent on the MOI. All four cell lines were seeded in 12 well plate at 5 × 10^4 ^cells/well 16–18 hrs before transduction. Viral vectors with known titre were added to each well at different MOI (MOI = viral titre/cell number). Medium was changed every day, with cells harvested 72 hrs after transduction, washed twice with PBS, and then examined by FACS analysis. Figure 4a shows that transduction efficiency of lentivectors was higher on the FRL-19 cell line than the other three cell lines. Transduction efficiency was 67.4% in FRL-19 cells, 33.1% in 293T cells, 23.1% in NIH 3T3 cells, and 8.7% in 293 cells at a MOI of 32. Generally, it was the higher the MOI, the higher the transduction efficiency (Fig 3B).

## Discussion

We showed in this study that a number of factors within the vector titration process, ie.: the volume of inoculum, the number of target cells, cell type and viability/susceptibility, vector exposure time for uptake and vector half life affected vector titre determination. We were also surprised to find that the volume of inoculum (with a constant virus concentration) played such an important role in the determination of transduction efficiency. It has been demonstrated that above the cell's surface in MoMLV based retroviral vector mediated gene transfer, a fluid layer of 0.1–1 mm thick remained stationary, and this layer is seen to be the major source of origin of the transducing elements. The large effects seen with non-agitated cultures in the present series of experiments with lentiviral vectors indicated some fundamental differences in the processes of the transduction pathways of MoMLV based retroviral vectors and lentivirally derived vectors. During the transduction process, the rate of collision between the virions and the surface of the target cells could be predicted from Brownian theory even when the viral suspension was being shaken continuously [22]. This appears to suggest that successful transduction depends on the concentration of virus and not the overall number of virions present, due to the layer effect. The fact that viral vector titre may vary from the transduction process and that the MOI was calculated based on the viral titre, suggested that different vector titres and MOIs could be generated from a single lentivector stock, making direct comparison of data difficult, especially when the difference in vector titre was as high as 50 fold. Therefore, the titre obtained this way obviously did not represent the true value of active vector concentration. Rather, it was grossly underestimated when commonly used cell lines were used as target cells for vector titration.

The viral stocks of most lentiviral vectors are generally produced from a 293 or 293T cell lines and the titre calculated by determining the number of foci (effect of the marker gene expression) produced in the cell line [23]. For example, if 100 μl of the vector suspension gives rise to 1 × 10^5 ^cells positive for a given marker gene expression, then the titre of the vector stock would be 1 × 10^6 ^TU/ml. When this vector stock is further used to transduce a new cell line, MOI is then determined by simply dividing the number of viral vector units added (ml added × TU/ml) by the number of target cells added (ml added × cells/ml). The average number of viral vector particles per cell in a transduction experiment could be less than 0.1 or more than 1000 depending upon how the experiment is designed. However, recent research showed that if MOI is too low, one may not get enough gene transfer and transgene expression [24]. If MOI is too high, the efficiency of gene transfer may not be very high, but many copies of transgene may integrate into the chromosomes of the target cells instead, thus causing chromosomal instability [24].

Employing MOI from 0–32, we demonstrated that efficient transduction of four different cell lines (293, 293T, NIH3T3, FRL19) resulted in a near liner relationship of MOI to transduction efficiency, the higher the MOI, the higher the transduction efficiency. This was somewhat surprising and contradicted traditional MoMLV based vectors, which showed an obvious plateau when the MOI was increased to about 3 [3]. The reason for this is unclear, but the fact that lentiviruses are more complicated retroviruses, having more sophisticated machinery for replication and integration than MoMLV, as well as that lentiviral vectors were exploiting the pseudotyped envelope (VSV-G utilises a different receptor), may probably explain the difference in gene transfer efficiency. The VSV-G envelope, binds to its target in cell membranes which are known to be phospholipids, such as phosphatidylcholine (PC) and phosphatidylserine (PS), (the receptors for VSV-G). PC is the most abundant membrane phospholipid while PS domains are present in much smaller quantity but bind more strongly and fuse faster with the VSV-G protein [25]. This issue is probably one of the most overlooked variables in vector transduction. Membrane phospholipid movement is highly dynamic. Its biosynthesis and degradation are very much dependent on cell type and positions in the cell cycle and/or metabolic activity. Also, the rate of degradation is rapid in G_1_, slows drastically during S phase, and picks up the pace again as cells exit mitosis and re-enters G_1_[26], which suggests that the cell cycle phase may be an important variable for VSV-G protein coated lentiviral transduction, and may contribute to the time dependence of the transduction efficiency observed in the present experiments. A further contribution to the volume effect may be increased cellular phospholipid uptake from the serum in the expanded volume of medium used for the delivery of the increased total vector or possibly enhanced phospholipid synthesis in a more generous nutritional environment. Cells double their phospholipid mass while maintaining the correct relative composition prior to cytokinesis [27]. Theoretically, during the intermitotic period the target cells will double the number of target binding sites for the viral vectors as well as allowing a period with the more favourable conditions (DNA synthesis) for integration. The amount of PC in the total membrane mass varies from 40–80% of the total P-lipid, depending on the cell type [27] and this variation may explain the discrepancies in transduction efficiencies observed with different cell lines using inocula of the same volume and titre of vector.

In the real world of gene transfer experiments, transduction conditions will be optimised to achieve the maximum efficiency. Generally, a high MOI is needed for satisfactory levels of gene transfer. Ideally, with a MOI of 2, every single cell might be expected to experience an average of two gene transfer events in a given transduction experiment, but probabilistic considerations of viral and vector-cell interactions ensure that this does not occur (i.e. only 67% of the cells would be "infected"). As seen in the current data, however, the efficiencies of transduction are very much less than the theoretical outcomes. Our study with lentiviral vector convincingly showed that the higher the MOI, the higher the efficiency of gene transfer and the level of gene expression. However, experiments employing MOI even greater than 1000 have still resulted in less than 100% of cells transduced [11,28,29] indicating the presence of unexplained variables in the cell dependence of the transduction process.

## Conclusions

MOI is only a useful term for predicting transduction efficiency under very carefully defined experimental conditions. The assumption is not valid that changes in any one of the variables shown to be important in the *in vitro *vector titration process will cause proportional changes in the magnitude of the transduction efficiency. It is thus evident that MOI is not applicable as a simply manipulable quantity in most gene therapy uses of the lentiviral vector system. Since clinical applications are an important outcome of gene transfer manipulations, and ultimately this may be done by *in vivo *delivery, the awesome task of evaluating the efficiency of transduction via this route will require considerable ingenuity. If MOI for lentiviral vector transduction has to be used for rigorous comparisons of data, then the specific experimental conditions for vector titration, with using the most sensitive cell lines, such as FRL 19, must be strictly observed for infectivity outcomes to be predictable.

## List of Abbreviations

CAEV, caprine arthritis/encephalitis virus; DMEM, Dulbecco's modified Eagle's medium; EGFP, enhanced green fluorescent protein; EIAV, equine infectious anaemia virus; FCS, Fetal Calf Serum; FITC, fluorescein isothiocyanate ; FIV, the feline immunodeficiency virus; HIV, Human Immunodeficiency Virus; JDV, Jembrana disease virus; MOI, Multiplicity of Infection; MoMLV, Moloney murine leukaemia virus; PC, phosphatidylcholine; PS, phosphatidylserine; VPC, vector-producing cell; VSV-G, Vesicular Stomatitis Virus G glycoprotein;

## Competing interests

None declared.

## Authors' contributions

BZ performed the use of MOI to predict gene transfer events in the four cell lines; PM performed titration of lentiviral vectors; HJ performed the statistics and Table 1. KE helped with design of the experiments in examining various conditions in *in vitro *transduction; GC provided some in BZ and HJ's work; M West provided advice on analysis of the data and manuscript writing; M Wei helped with the design and day to day supervision of all the experiments, assisted with analysing the data and prepared and prove read the manuscript.
